# Transcriptome Analysis of Chinese Cabbage Provides Insights into the Basis of Understanding the Lignin Affected by Low Temperature

**DOI:** 10.3390/genes13112084

**Published:** 2022-11-10

**Authors:** Yun Dai, Shaoxing Wang, Wenyue Huang, Ze Li, Shifan Zhang, Hui Zhang, Guoliang Li, Zhiyuan Fang, Rifei Sun, Fei Li, Shujiang Zhang

**Affiliations:** Institute of Vegetables and Flowers, Chinese Academy of Agricultural Sciences, Beijing 100081, China

**Keywords:** low temperature, Chinese cabbage, RNA-seq, WGCNA, lignin biosynthesis, *BrCOMT* genes

## Abstract

Chinese cabbage, which is a cold season crop, can still be damaged at an overly low temperature. It is crucial to study the mechanism of the resistance to low temperature of Chinese cabbage. In this study, the Chinese cabbage ‘XBJ’ was used as the material, and nine different low temperatures and control samples were treated. Using RNA-seq and lignin content determination, we analyzed 27 samples, and the stained sections of them were observed. A total of 8845 genes were screened for the WGCNA analysis, yielding 17 modules. The GO and KEGG analyses of the modules was highly associated with a low-temperature treatment. The pathways such as ‘starch and sucrose metabolism’ and ‘plant hormone signal transduction’ were enriched in modules related to low temperature. Interestingly, L-15DAT-associated MEcoral2 was found to have 14 genes related to the ‘lignin biosynthetic process’ in the GO annotation. The combination of the determination of the lignin content and the treatment of the stained sections showed that the lignin content of the low-temperatures samples were indeed higher than that of the control. We further explored the expression changes of the lignin synthesis pathway and various genes and found that low temperature affects the expression changes of most genes in the lignin synthesis pathway, leading to the speculation that the lignin changes at low temperature are a defense mechanism against low temperatures. The 29 *BrCOMT* gene sequence derived from the RNA-seq was non-conserved, and eight *BrCOMT* genes were differentially expressed. This study provides a new insight into how lignin is affected by low temperature.

## 1. Introduction

Chinese cabbage (*Brassica rapa* L. ssp. *pekinensis*) is a traditional vegetable with a long history of use in China [1]. Although Chinese cabbage is a cool season crop, an overly low temperature or an extended period of low temperature would still cause harm to Chinese cabbage. In the growth cycle of plants, the reproductive growth stage is the one that is the most vulnerable to temperature, and it includes the formation of reproductive organs, flowering, fruit bearing, and seed maturation. Once they have encountered a low temperature, the yield of the crops would be seriously affected [2,3]. A low temperature would increase the activity of the antioxidant enzymes [4], malondialdehyde [5], protein [6], etc. Meanwhile, Chinese cabbage would derive metabolites such as anthocyanin [1] and melatonin [7] when it encounters a low-temperature environment, and many genes related to cold tolerance such as *CBFs*, *ICE1,* and *WRKYs* have been identified [8,9].

Under a drought stress, the lignin content in known to increase, and this has been shown in, for example, eucalyptus (*Eucalyptus robusta*) [10]. The expression level and lignin content of the lignin synthesis in maize (*Zea mays*) increased significantly [11]. When a plant is infected with a pathogen, the cell wall accumulates large amounts of lignin [12,13]. Lignin-synthesis-related genes and disease-related genes were found to be significantly upregulated in rice varieties [14,15]. *ZmCCoAOMT2* in maize was found to be associated with a resistance to a variety of pathogens, which are potentially involved in the metabolic pathways that are involved in lignin biosynthesis and in regulating programmed cell death [16]. Studies have proven that the increased content of lignin in plant tissues can enhance their cold resistance. For example, lignin deposition in Norway spruce was negatively correlated with temperatures from early September to late October [17]. Ex vitro poplar (*Populus tremula* × *Populus tremuloides* L. cv. Muhs1) seedlings that are grown at 10 °C showed an increased lignin content [18].

At present, RNA-seq technology can be applied to the study of various traits in various plants, such as using RNA-seq to study the identification of *Lycium chinense* biosynthesis genes and the accumulation of phenylpropanoids [19]. RNA-seq was used to investigate the protection of anthocyanins against *Begonia semperflorens* at low temperatures [20], and RNA-seq was used to study the regulation of elephant grass lignin synthesis [21]. In this study, Chinese cabbage was studied at a low temperature by the RNA-seq method, and the lignin synthesis pathway was further explored at a low temperature.

In this study, the Chinese cabbage ‘XBJ’ was used as planting material, and it was grown under four different low temperatures, and these were compared with five control growths. Twenty-seven samples had RNA-seq applied to them, wherein the lignin content was determined and analyzed, and the stained sections were observed. Combined with the GO, KEGG, WGCNA, and qRT-PCR correlation analyses, the changes to the lignin synthesis pathway genes in the Chinese cabbage under the low-temperature treatment were identified, thus providing valuable genomic data for the molecular mechanism of low-temperature resistance in Chinese cabbage.

## 2. Materials and Methods

### 2.1. Plant Materials and Treatments

The highly inbred Chinese cabbage ‘Xiao Baojian’ (XBJ) was provided by the breeder, Shujiang Zhang. The plants were initially grown at 25 ± 2 °C under natural light for 32 days. Subsequently, the seedlings were then divided into two parts and treated at 25 °C (16/8 h light/dark photoperiod and 150 µmol m^−2^ s^−1^ light intensity) for 0, 15, 25, 35, and 45 days (N-0DAT, N-15DAT, N-25DAT, N-35DAT, and N-45DAT, respectively; days after normal treatment) at a normal temperature, and at 4 °C (16/8 h light/dark photoperiod and 150 µmol m^−2^ s^−1^ light intensity) for 15, 25, 35, and 45 days at a low temperature (L-15DAT, L-25DAT, L-35DAT, and L-45DAT, respectively; days after low-temperature treatment). The third fully expanded leaves from the center of the plants were sampled from each treatment. After the sampling, they were immediately frozen in −80 °C liquid nitrogen for the subsequent RNA-seq experiments and quantitative real-time polymerase chain reaction (qRT-PCR) analyses.

### 2.2. Determination of Lignin Content

The lignin content was measured via the micromethod using the Plant Lignin Content Detection Kit (Beijing Solarbio Science and Technology Co., Ltd., Beijing, China). A sample mass of 0.005 g was used for the calculation. We measured the absorbance value A at 280 nm, recording it in the A measuring tube or the A blank tube, and then, we calculated ∆A = A measuring tube − A blank tube. Then, we calculated the lignin content: lignin content (mg/g) = 2.184 × ∆A ÷ 0.005. All of the operations were conducted with three repeats.

### 2.3. RNA Extraction and Illumina Sequencing

The samples from each period were stored at −80 °C for the RNA extraction. The leaf samples were used for the total RNA extraction, and an RNA extraction kit (Vazyme, Nanjing, China) was used following the manufacturer’s protocol. The RNA concentrations and its quality were verified using an Agilent 2100 Bioanalyzer (Agilent Technologies, Santa Clara, CA, USA). A total amount of 1 µg RNA per sample was used as the input material for the RNA sample preparations. A total of 27 cDNA libraries were constructed and sequenced using NEBNext^®^Ultra™ RNA Library Prep Kit for Illumina (New England Biolabs, Ipswich, MA, USA) following the manufacturer’s recommendations. The Illumina raw data were submitted to the Sequence Read Archive (SRA) database at NCBI under the BioProject number PRJNA763246 under the following link: https://www.ncbi.nlm.nih.gov/bioproject/PRJNA763246, (accessed on 9 September 2021).

### 2.4. RNA-Seq Analysis

The expression level of the unigenes was estimated using Cufflinks v2.2.1 [22], and the relative abundance in each sample was normalized to the fragments per kilobase per million reads (FPKM). DESeq2 [23] was used to perform the differential expression analysis of two samples. The Benjamini–Hochberg approach [24] was used to control the false discovery rate to adjust the obtained *p*-value. The genes with adjusted *p*-values of <0.01 which were found by DESeq2 were designated as differentially expressed.

The unigene functions were annotated on the basis of the Gene Ontology (GO) enrichment analysis using the GOseq R software [25]. The KOBAS software [26] was used to test the statistical enrichment of the genes in the Kyoto Encyclopedia of Genes and Genomes (KEGG) pathways. The GO and KEGG drawings were obtained through the BMKCloud website (www.biocloud.net, accessed on 9 September 2021).

### 2.5. Gene Co-Expression Network Analysis and Visualization

The RNA-seq data were analyzed to construct the gene co-expression networks using the R package WGCNA [27]. On the basis of the software default standard, the co-constructed genes were screened from the ‘XBJ’ RNA-seq data for the analysis. The module eigengenes were intended to describe the most common gene expression models in each module. The module eigengenes summarized the module overview and feature data as they were the first major component of the expression matrix. Pearson’s correlation coefficients were used to calculate the correlation between the modular eigengenes and each treatment period of ‘XBJ’. The correlation is reflected by the depth of the color in the heatmap.

### 2.6. Histochemistry and Autofluorescence Microscopy

The lignified leaves were identified in the sections through histochemistry and fluorescence microscopy. The steps were: ① the leaves were fixed in FAA (50 mL of 40% formaldehyde, 50 mL of glacial acetic acid, and 90 mL of 50% ethanol) for 24 h, dehydrated with ethanol, and then, they were embedded in paraffin; ② we took out the tissue in the fixative and trimmed it into thick slices with a thickness of 0.2–0.3 μm, pre-cooled the Carnoy fixative for 30 min under negative pressure, and then, we fixed it at low temperature for 1–3 h, and then, we directly transferred it to 75% ethanol for normal dehydration and embedding; ③ after dewaxing the sections, we put them in a safranin staining solution for 1–2 h; ④ we rinsed the sections with distilled water for 4–10 s to remove the excess dye; ⑤ we added 75%, 85%, and 95% alcohol for a gradient decolorization, and they were decolorized for 3–5 s each time; ⑥ we transferred them to a fast green staining solution for fast green staining, and then, we transferred them out after staining them for 15 s, and we quickly rinsed the sections with 95% ethanol; ⑦ we used absolute ethanol for the elution, and a treatment was applied twice for 3 min each time; ⑧ we used xylene transparent and neutral gum for the sealing. To visualize the lignin autofluorescence, the tissue sections with lignin were visualized by fluorescence microscopy under UV excitation.

### 2.7. COMT Gene Sequence Alignment, Phylogenetic Analyses, and Gene Structure Determination

The caffeic acid O-methyltransferase (*COMT*) gene sequences of Arobidopsis *thaliana* (*A. thaliana*) were obtained from the TAIR data center, and they were downloaded (http://www.arabidopsis.org/, accessed on 9 September 2021), and the *COMT* gene sequences of ‘XBJ’ were obtained in this RNA-seq data using the sequences of ‘XBJ’ and *A. thaliana* using Mega 7.0 software, which was used to construct the evolutionary tree using the NJ method, for which the bootstrap method of the phylogenetic tree was set to 1000, whereas other parameters that were used were the default values [28]. Protein conserved motifs of the *COMT* family of *A. thaliana* and ‘XBJ’ were analyzed using the MEME online tool (MEME—submission form (meme-suite.org, accessed on 9 September 2021)) with a maximum search value of 10. TBtools was used to analyze the members of the gene family by comparing the CDS sequences of the *COMT* genes [29]. The 2000 bp region upstream of the ATG of *COMT* genes were extracted, and the cis-acting elements in the promoter were analyzed using the PlantCARE software [30].

### 2.8. qRT-PCR Analysis and Statistical Analysis

The RNA that was extracted from each sample was reverse-transcribed into cDNA using HiScript III All-in-One RT SuperMix Perfect (Vazyme, Nanjing, China), and a qRT-PCR was performed using Taq Pro Universal SYBR qPCR Master Mix (Vazyme, Nanjing, China) using a CFX-96 Real-time System (BIORAD, Hercules, CA, USA). The primer design was completed using Primer v5.0 (Table S1). *Actin* was considered as the internal control, and the gene expression data were analyzed using the 2^−ΔΔCt^ method [31]. SPSS v19.0 (SPSS, Chicago, IL, USA) was used to conduct a one-way analysis of variance (ANOVA) with a Duncan’s multiple range post hoc test, and there was a significance threshold of *p* < 0.05. The results were visualized using Sigmaplot v10.0 (Systat Software Inc., San Jose, CA, USA).

## 3. Results

### 3.1. Identification and Characterization of RNA in Chinese Cabbage at Low-Temperature Stage

In total, 1,183,051,830 total reads, 591,525,915 clean reads, and 176.89 Gb clean data from 27 samples were obtained through the RNA-seq analysis. The percentage of the Q30 base ranged from 91.50 to 94.73%, and the average GC content was 46.22% (Table S2). The clean reads of each sample were compared with the designated *B*. *rapa* reference genome (v 3.0) [32], and the comparison efficiency ranged from 70.57 to 90.87% (Table S2). We performed correlation tests between the samples using Pearson’s correlation coefficients. The generated cluster dendrogram was used for the overall correlation of ‘XBJ’ across the transcriptomes of the different treatments (Figure S1A). The similarity test between the samples was a principal component analysis (PCA), and by using the first principal component (PC1) and the second principal component (PC2), the similarity between the replicates of each sample was analyzed by performing dimensionality reductions (Figure S1B). The number of transcripts between the samples also showed high similarity, with a log_10_FPKM volatility that was similar across all of the samples (Figure S1C).

**Figure 1:**
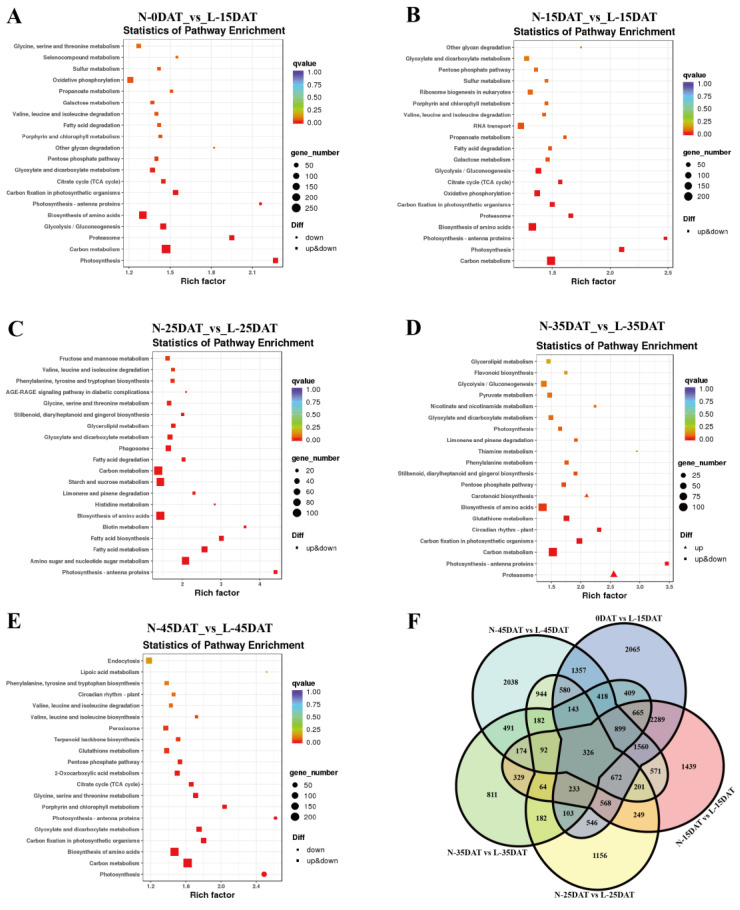
Identification and functional characterization of the differentially expressed genes (DEGs) between different samples. KEGG enrichment analysis of the DEGs for: (**A**) N-0DAT_vs_L-15DAT_KEGG, (**B**) N-15DAT_vs_L-15DAT_KEGG, (**C**) N-25DAT_vs_. L-25DAT_KEGG, (**D**) N-35DAT_vs_L-35DAT_KEGG, and (**E**) N-45DAT_vs_L-45DAT_KEGG. (**F**) Venn diagram depicting the DEG number between the five compared groups of samples.

The most enriched KEGG terms among the DEGs that were detected for all of the compared samples were photosynthesis-related and ‘carbon metabolism’. Among them, the gene number of ‘carbon metabolism’ accounted for the largest proportion, and the second one was the ‘biosynthesis of amino acids’ (Figure 1A–E). There were 326 DEGs in the comparative analysis of the DEGs among the five groups of samples (Figure 1F). 0DAT vs. L-15DAT was the group with the largest number of DEGs, indicating that many genes changed the most in the early stage of the low-temperature condition, and they may have slowly adapted to the low-temperature environment in the later stage.

### 3.2. Gene Co-Expression Network Construction of Low-Temperature Environment Found That It Is Related to the Lignin Biosynthetic Process

**Figure 2:**
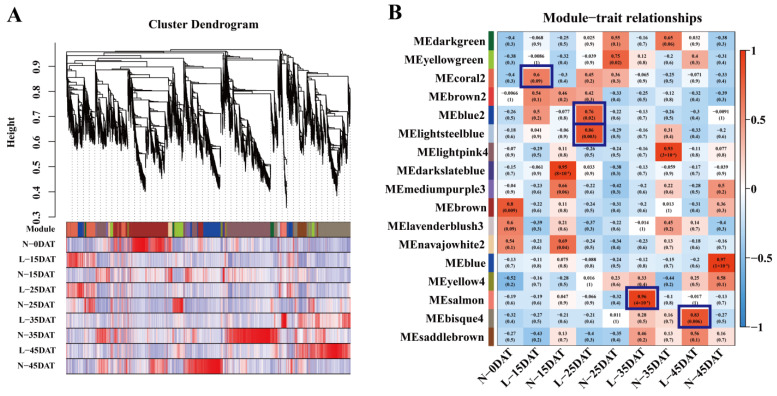
WGCNA analysis of ‘XBJ’ at different low temperatures. (**A**) Hierarchical cluster trees showed the co-expression modules identified by WGCNA. (**B**) Co-expression modules by WGCNA. Relationships between modules (left) and traits (bottom). Red and blue represent positive and negative correlations, respectively, with coefficient values and *p*-values.

The transcriptome data from each treatment period of ‘XBJ’ were analyzed to filter out the low-abundance and low-variability genes. A total of 8845 genes were screened out (Table S3) and imported into the WGCNA software package for the analysis. The transcriptome data analysis was performed at each epoch for the WGCNA analysis. Each tree branch forms a module, and each leaf in the branch represents a gene, as shown in the hierarchical clustering tree (Figure 2A). Then, the tree in the dendrogram was cut into modules (clusters). The genes (modules) were identified on the basis of their correlation with the low-temperature treatment. As shown in the dendrogram, the WGCNA analysis yielded 17 modules, which are distinguishable by them having different colors (Figure 2B, Table S3). Whether the correlation was positive or negative, as well as the magnitude of the correlation, indicated the degree of correlation with the target genes that were screened for in the transcriptome data for that period (Figure 2B).

The module–trait relationships (MTRs) were different for each low-temperature treatment. These modules contained positively and negatively related genes, and their expression levels changed at different treatments. We further observed several modules that were highly correlated with the low-temperature treatments: MEcoral2 (r = 0.6, *p* = 0.09) in L-15DAT; MEblue2 (r = 0.76, *p* = 0.02) in L-25DAT; MElightsteelblue (r = 0.86, *p* = 0.003) in L-25DAT; MEsalmon (r = 0.96, *p* = 4 × 10^−5^) in L-35DAT; MEbisque4 (r = 0.83, *p* = 0.006) in L-45DAT.

Subsequently, we further analyzed and explored these five modules. There were two modules that were highly correlated with L-25DAT, MEblue2 (r = 0.76, *p* = 0.02) and MElightsteelblue (r = 0.86, *p* = 0.003), and we performed GO, KEGG, and eigengene expression analyses on them (Figures S2 and S3). In MEblue2, ‘copper ion binding’, ‘apoplast’, and ‘cell wall organization’ were the three most enriched terms for the GO annotations for the molecular function, cellular component, and biological process (Figure S2A) respectively, and the KEGG pathway was enriched in ‘amino sugar and nucleotide sugar metabolism’ and ‘biosynthesis of amino acids’, etc. (Figure S2B). In MElightsteelblue, the gene annotations were relatively simple, focusing on various ‘dehydrogenase activities’ (Figure S3A). The eigengene expressions of MEblue2 and MElightsteelblue were most prominent in L-25DAT (Figures S2C and S3C). ‘Electron carrier activity’, ‘anchored component of membrane’, and ‘carbohydrate metabolic process’ were enriched in L-35DAT MEsalmon GO annotations (Figure S4A), and that of the KEGG enrichment analysis was ‘starch and sucrose metabolism’ (Figure S4B). MEbisque4 was highly correlated with L-45DAT; the GO terms were mainly enriched in ‘molecular function’ and ‘biological process’, and particularly, ‘sequence-specific DNA binding’ and ‘response to wounding’, while KEGG-enriched one was ‘plant hormone signal transduction’ (Figure S5A,B). The eigengene expressions of MEsalmon and MEbisque4 were mainly highly expressed in L-35DAT and L-45DAT (Figures S4C and S5C).

**Figure 3:**
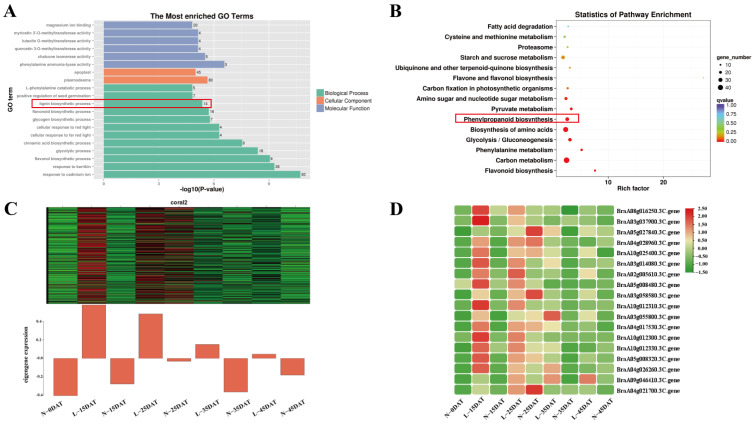
MEcoral2-related genes and their annotations. (**A**) Significant GO terms in MEcoral2. (**B**) Significant KEGG terms in MEcoral2. (**C**) Eigengene expression during each treatment period in MEcoral2; heatmap from green to red for expression from high to low, respectively. (**D**) Heatmap of lignin-synthesis-related gene expression in MEcoral2.

Interestingly, we found that MEcoral2, which is related to L-15DAT, has 14 genes that are associated with the ‘lignin biosynthetic process’ in the GO annotation, and it was also enriched in the KEGG annotation of ‘phenylpropanoid biosynthesis’, which is an essential metabolic pathway for lignin synthesis (Figure 3A,B). The expression levels of 14 lignin-related synthetic genes were essentially higher than those of the control at the same time in the low-temperature environment (Figure 3C,D).

### 3.3. Lignin Biosynthetic Process Analysis and Related Genes Identification

**Figure 4:**
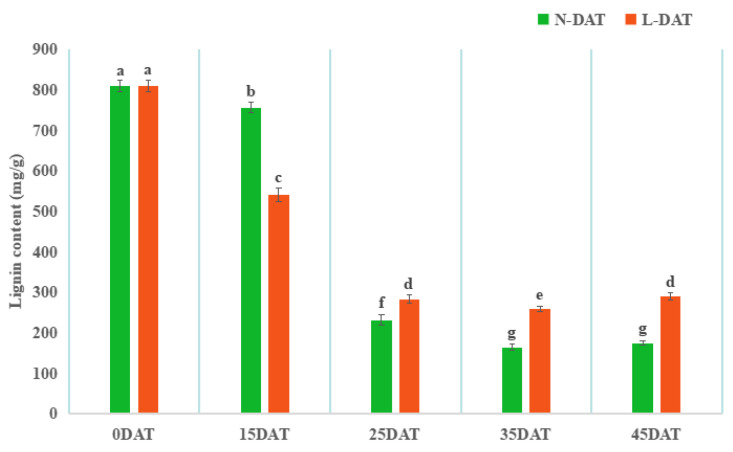
Total lignin content in ‘Xiao Baojian’ (XBJ) in different treatments. Error bars represent the SE (*n* = 3). Values with the same letter were not significantly different at *p* < 0.05. N-DAT represents days after normal treatment, and L-DAT represents days after low-temperature treatment.

For the drying samples that were obtained (N-0DAT, N-15DAT, N-25DAT, N-35DAT, and N-45DAT; and L-15DAT, L-25DAT, L-35DAT, and L-45DAT), we first carried out the content determination of the lignin (Figure 4). The content of lignin in the untreated state (0 DAT) was the highest. In comparison with that of the control, the low-temperature condition would reduce the lignin more at the beginning. Under the continuous influence of a low temperature, the lignin content of the control decreased the most. It showed that, to a certain extent, the accumulation of lignin would be stabilized in the latter stage of a low-temperature environment to cope with the damage that is caused by a low temperature.

**Figure 5:**
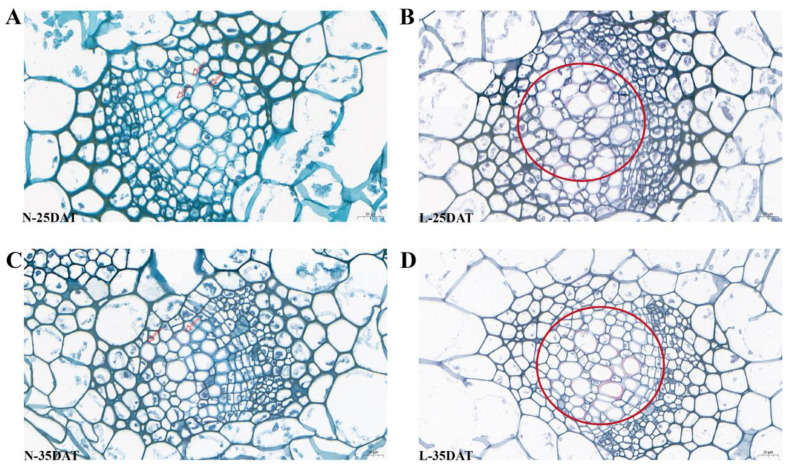
Safranin staining of lignin in ‘XBJ’ leaves at four treatments. (**A**) N-25DAT of ‘XBJ’ × 40; (**B**) L-25DAT of ‘XBJ’ × 40; (**C**) N-35DAT of ‘XBJ’ × 40; (**D**) L-35DAT of ‘XBJ’ × 40.

We stained the leaves at the stages N-25DAT, L-25DAT, N-35DAT, and L-35DAT with a safranin staining solution. It was clear that the lignin in the leaves at a low temperature was more obvious than it was in the control period (Figure 5); the overall leaf colors of L-25DAT and L-35DAT were reddish, the lignin in the cells was more enriched (Figure 5B,D, circles shown), and N-25DAT and N-35DAT only had some lignin, sporadically (Figure 5A,C, arrows shown).

**Figure 6:**
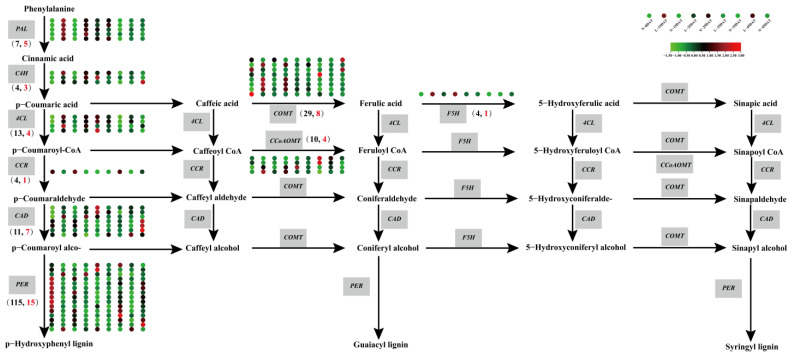
Lignin synthesis pathway of Chinese cabbage under low temperature. The nine circles of the heat map correspond to nine treatments. The gray boxes indicate different genes. The total number of genes in the brackets indicate black font, and the number of DEGs indicate red font.

The focus of the present study was on identifying the underlying mechanisms that are associated with the differences in Chinese cabbage at a low temperature. Phenylalanine ammonia-lyase (PAL) functions at the beginning of the phenylpropanoid pathway, catalyzing the formation of cinnamic acid and p-coumaroyl-CoA, after which the phenylalanine pathway divides into two branches: the phenylalanine metabolic pathway and the flavonoid metabolic pathway; the synthesis of lignin is synthesized by the phenylalanine metabolism pathway (Figure 6, Table S4). *PAL* and cinnamate 4-hydroxylase (*C4H*) are phenylpropanoid pathway genes, and they are also the starting point for the synthesis of lignin. In this study, seven *PALs* and four *C4Hs* were found, but five *PALs* and three *C4Hs* genes were DEGs under the low-temperature condition and in the control. The expression levels of these DEGs in the low-temperature samples were higher than those in the control, indicating that a low temperature had a significant impact on the phenylalanine metabolism pathway. There were a total of four 4-coumaric acid coenzyme A (CoA) ligases (*4CLs*), seven cinnamyl alcohol dehydrogenase (*CADs*), eight *COMTs*, one ferulate 5-hydroxylase (*F5H*), four caffeoyl-CoA O-methyltransferase (*CCoAOMTs*), and fifteen peroxidases-synthesized (*PERs*-synthesized) three lignins, namely, p-hydroxyphenyl lignin (H type), guaiacyl lignin (G type), and syringyl lignin (S type). The expression levels DEGs of the *4CLs* were higher under the low-temperature treatment, and the four DEGs of *COMTs* had higher expression levels at the L-15DAT and 25DAT periods. Only one *CCoAOMT* gene had higher expression levels at L-15DAT. The expression level of the *F5H* gene in the three low-temperature periods (L-15DAT, L-25DAT, and L-35DAT) was higher it was for the control. The enzyme CAD catalyzes the conversion of cinnamic aldehyde to cinnamyl alcohol, which is the final step in the synthesis of lignin monomer alcohols. *PER* is the final gene for the synthesis of the three lignins. In this study, the expression of one *CAD* was higher in L-15DAT and L-25DAT, and the expression of two *PERs* were higher in the low-temperature period than they were in the control. It could be seen that the difference between these genes under the low temperature and in the control showed that the low temperature did affect the synthesis of lignin, and thus, maybe the change of lignin was a defense measure for Chinese cabbage to cope with the low temperatures.

### 3.4. COMT Gene Sequence and Conserved Analysis

Caffeic acid O-methyltransferase (COMT) is an important protein that participates in lignin synthesis and is associated with the ratio of G-/S-type lignin in plants. We identified 29 *COMT* genes by RNA-seq, and we named them *BrCOMT1* to *BrCOMT29*.

**Figure 7:**
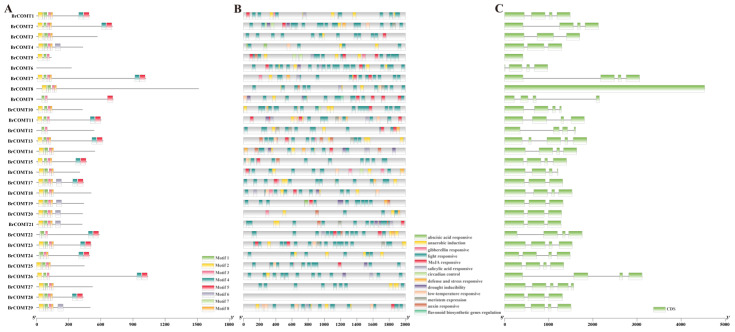
Conserved analysis of the 29 *BrCOMT* genes. (**A**) Amino acid sequence analysis. (**B**) Analysis of cis-acting elements of promoters. (**C**) Exon and intron analysis.

To understand the structural diversity and structural characteristics of the 29 *BrCOMT* genes in Chinese cabbage, a motif analysis of the 29 *BrCOMT* amino acid sequences was carried out by the MEME program. A total of eight motifs were identified in the *BrCOMT* genes, and the numbers and types of motifs in the *BrCOMT* genes were significantly different between the groups, which indicated that the amino acid sequences and gene structures of the *BrCOMT* family were not conserved and had functional differences. Only the *BrCOMT17* gene (BraA06g016590.3C) contained eight motifs; the *BrCOMT1, BrCOMT23,* and *BrCOMT28* genes (BraA01g006200.3C, BraA07g026990.3C, and BraA08g027460.3C) contained seven motifs. Most of the other *BrCOMT28* genes contained six or five motifs (Figure 7A). In addition, to better study the gene expression and transcriptional regulation, the cis-acting elements in 29 *BrCOMT* genes’ promoter regions were analyzed by TBtools (Figure 7B, Table S5). There were 173 hormone-related cis-acting elements, including abscisic-acid-responsive, auxin-responsive, gibberellin-responsive, MeJA-responsive, and salicylic-acid-responsive. There were 337 light-responsive elements, which were the most common type. A total of 135 cis-acting elements were related to stress, such as anaerobic conditions, drought, and low temperatures. In addition to these, there were the ‘circadian control’, ‘flavonoid biosynthetic genes regulation’, and ‘meristem expression’ types. It could be seen that the *BrCOMT* genes may not only play a key role in lignin synthesis, but they may also play a certain role in hormone regulation and stress. TBtools also analyzed and identified the structure of the exons and introns of the *BrCOMT* genes (Figure 7C). Most *BrCOMT* genes contain four exons and three introns. Only *BrCOMT13* (BraA02g014110.3C) and *BrCOMT27* (BraA08g017380.3C) contain five exons and four introns.

**Figure 8:**
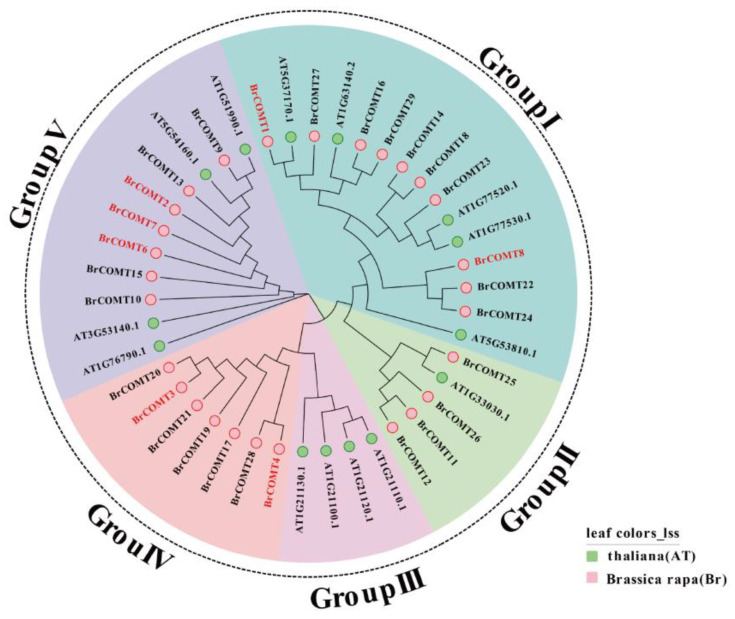
Phylogenetic analysis of 14 and 29 COMT protein sequences from *A. thaliana* and Chinese cabbage, respectively.

To study the evolutionary relationships of the *BrCOMT* genes, a total of 14 and 28 protein sequences from *A. thaliana* and Chinese cabbage (except *BrCOMT5*) were used to construct a phylogenetic tree (Figure 8). Combined with 14 genes in *A. thaliana*, 42 genes were mainly divided into five categories, namely, group I, group II, group III, group IV, and group V. Group I was the largest group with 15 genes and next, was group V, with 11 genes. The seven genes in Group IV belonged to the *BrCOMT* genes, and these seven genes independently form a group. These results indicated that the *COMT* genes of Chinese cabbage and *A. thaliana* were different.

### 3.5. qRT-PCR Validation of COMT Genes

**Figure 9:**
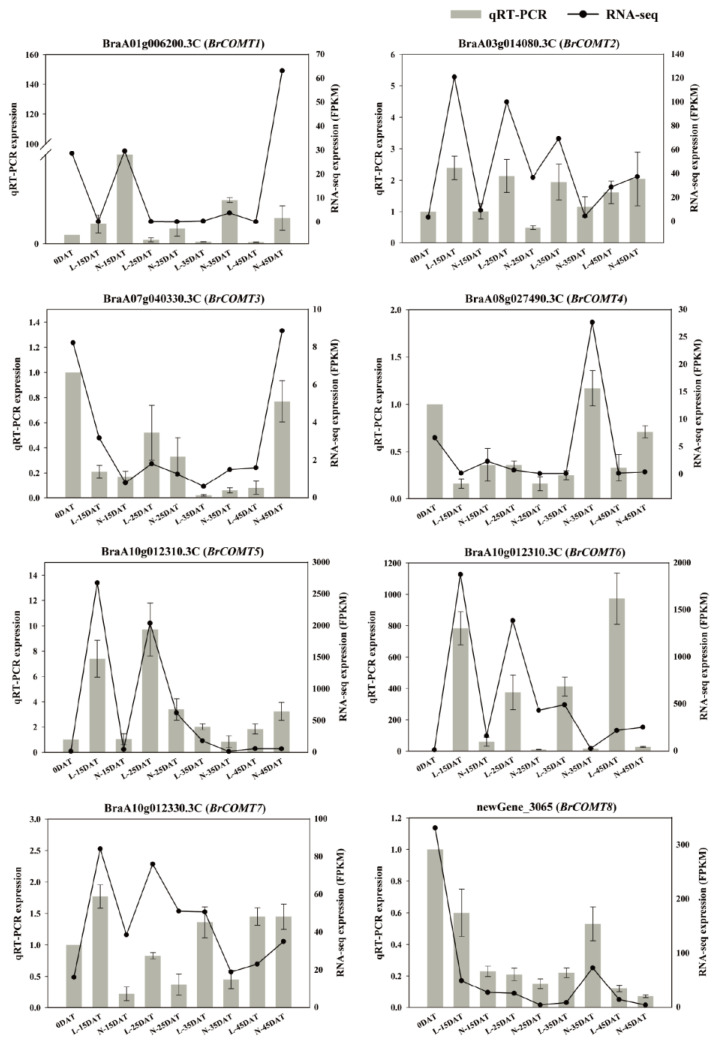
qRT-PCR was performed using 8 DEGs of *COMT* genes. Bar and line graphs represent the qRT-PCR and RNA-seq data, respectively. Data are presented as the mean ± standard error (SE).

The transcription levels of eight DEGs of *COMT* genes were evaluated using qRT-PCR. The RNA-seq and qRT-PCR results were consistent during the low-temperature process (Figure 9), indicating the reliability of the high-throughput transcriptome sequencing. It can be seen from the intuitive result map that most the DEGs of *COMT* were more expressed in the low-temperature environment than they were in the control environment, indicating that the *COMT* genes were obviously affected by the low temperature, thus further affecting the lignin synthesis at a low temperature and laying the foundation for subsequent studies.

## 4. Discussion

Lignin is one of the main components of the cell walls. It is involved in an organism’s response to abiotic stresses (drought, waterlogging, salinity, high temperature, low temperature, heavy metal stress, shading, and high concentrations of CO_2_, etc.). Thus far, there has not been any information that has been provided on the effects of a low temperature on the lignin of Chinese cabbage. Although Chinese cabbage is a cool season crop, an overly low temperature or an extended period of a low temperature would still cause harm to the Chinese cabbage. We performed low-temperature and control treatments on the Chinese cabbage ‘XBJ’ and applied RNA-seq.

We executed a WGCNA analysis of the RNA-seq data and obtained 17 clusters that are associated with the low-temperature and control conditions. Differential genes related to lignin synthesis were enriched in MEcoral2 that were associated with the low-temperature treatment (4 °C, same as below) that occurred for 15 days (Figure 2 and Figure 3). Lignin is an essential secondary metabolite that is mainly derived from the metabolic pathway of phenylpropanoid in plant cells [33]. We executed GO and KEGG analyses on MEcoral2 and found that it was enriched in the ‘lignin biosynthetic process’ and ‘phenylpropanoid biosynthesis’ (Figure 3A,B). The genes in MEcoral2 were expressed more under the low-temperature treatment (Figure 3C), and the genes related to lignin synthesis were also more expressed under the low-temperature treatment (Figure 3D). It could be seen that a low temperature can indeed cause changes in the lignin synthesis pathway in the Chinese cabbage. In order to verify the lignin content at the low-temperature and in the control conditions, we measured the lignin content of the samples in each treatment period. We found that a low temperature reduced the lignin content of the Chinese cabbage, but as the treatment time progressed, the lignin content in the low-temperature condition was still slightly higher than it was in the control condition (Figure 4). Similarly, the safranin solution staining was performed on the leaves under the low-temperature and control staining conditions, resulting in a significantly greater lignin enrichment area under the low temperature condition than that which was seen under the control staining condition (Figure 5), which is also consistent with the lignin content that is shown in Figure 4. The results of these experiments were similar to those of previous studies, wherein ex vitro poplar (*P*. *tremula* × *P. tremuloides* L. cv. Muhs1) seedlings that were grown at 10 °C showed an increase in the lignin content [18], and the lignin contents in tuberous roots increased 1.1–1.3-fold under a 4 °C storage condition when they were compared to the storage condition at 13 °C [34].

A low temperature would change the gene expression and plant metabolism, thus affecting many biological functions. Lignin that is deposited in plants could resist the damage that is caused by a low-temperature stress by strengthening the cell wall [35]. We plotted the lignin synthesis pathway and expression level of the Chinese cabbage ‘XBJ’ in the low-temperature and the control conditions (Figure 6). The biosynthesis of the lignin monomer underwent a series of enzymatic reactions. At least nine enzymes catalyzed these reactions. PAL and C4H are phenylpropanoid pathway genes, and these are the starting point for the lignin synthesis. Five *PALs* and three *C4Hs* genes were differentially expressed at the low temperature, which proved that the low temperature affected the lignin at the beginning. Studies have demonstrated that Chilling and Freezing 1 (*TCF1*) could be rapidly induced to activate blue-copper-binding PROTEIN (BCB) transcription under low-temperature conditions (−8 °C or −10 °C for 2 h), and then, *PAL1/3/4* expression can be stimulated to maintain the lignin accumulation [36]. The expression of the C4H gene was also changed when it was under abiotic stresses. Gray poplar (*Populus × Canescens*) increased the *C4H* expression after flooding [37]. The lignin biosynthesis pathway proteins DcC4H rose in the fleshy roots of carrots that were under elevated CO_2_ stress [38]. 4-Coumarate-CoA ligase (4CL) is a key turning point enzyme in the lignin synthesis process, whereas cinnamoyl-CoA reductase (CCR), CAD, and PERs synthesize H-type lignin. It was proven that the chilling condition during the postharvest storage of *Eriobotrya japonica* resulted in increased expression levels of *PAL*, *4CL*, and *CAD*, as well as an increased lignin content [39]. The other side of H-type lignin synthesis was affected by the enzymes such as COMT, CCoAOMT, and F5H, which would further form the G-type and S-type lignins. After 75 days of a 14.5 °C treatment of sugarcane (*Saccharum spp*.), the downregulation of *ShCAD2*, *ShCOMT1*, and *ShCCoAOMT1* in the sugarcane stem resulted in the reduction in the lignin content, while the upregulation of *ShF5H* in the pith increased the lignin content [40]. In our study, the expression levels of *COMT*, *CCoAOMT*, and *F5H* increased in the early stage of the low-temperature condition, and they decreased slightly as the low-temperature condition progressed. These results indicated that the change of the lignin synthesis gene at the early stage of the low-temperature condition was an emergency defense mechanism against low temperatures, that was employed to protect the survival of the Chinese cabbage to a certain extent.

Previous studies have shown that the *COMT* gene can be induced by cold, H_2_O_2_, and salt conditions [41], while under drought stress, the expression levels of some *COMT* family genes in *Brassica napus* were higher than that they were under the non-stressed conditions [42], and the expression of the *COMT* gene in Ligusticum could be significantly induced by cold and drought [43]. Here, we performed the sequence and conserved analysis of the 29 *COMT* genes which were obtained by RNA-seq, and we named them *BrCOMT1* to *BrCOMT* 29. The *COMT* genes in the Chinese cabbage and the *COMT* genes in *A. thaliana* were different, indicating that the *COMT* in Chinese cabbage undergoes differentiation events (Figure 8). A total of eight motifs were identified in twenty-nine *BrCOMT* genes; a few *BrCOMT* contained eight motifs, while most of them contained six or five motifs (Figure 7A), indicating that the amino acid sequence and structure of *BrCOMT* were not conserved. In our study, most of the *BrCOMTs* had three introns, and the members generally had similar exon–intron structures. However, *BrCOMT8* did not contain introns and had only one long exon region (Figure 7C), indicating that the evolution of the *COMT* gene family may be closely related to the diversification of the gene structures. Similar results have been obtained in other gene families [44]. In our study, cis-acting elements that are related to stress, such as anaerobic conditions, drought, low temperatures, and some hormone-related responses, were present in some of the *BrCOMT* promoter sequences (Figure 7B). In our study, eight *COMT* genes (*BrCOMT1* to *BrCOMT8*) were differential genes at a low temperature. We performed a qRT-PCR to further verify that a low temperature can cause the differential expression of *COMT* in Chinese cabbage and that it has an impact on lignin synthesis (Figure 9).

## 5. Conclusions

In this study, we examined the low temperature and control of Chinese cabbage ‘XBJ’, and we performed a WGCNA analysis. We found that the enrichment of the ‘lignin biosynthetic process’ and ‘phenylpropanoid biosynthesis’ in MEcoral2 is associated with L-15DAT. We further determined the lignin content and section staining on each sample and found that with the low-temperature treatment, the cold samples had a higher lignin content than the control samples did during the same period. We explored the expression changes of the lignin synthesis pathway and the expression of each gene, and we found that the low temperature affected the expression changes in most of the genes in the lignin synthesis pathway, and we presume that the lignin changes in the low-temperature condition were a defense mechanism against low temperatures. The *COMT* genes have essential roles in lignin synthesis and abiotic stress. In this study, the 29 *BrCOMT* genes which were obtained from sequencing were explored and found to be different in their sequences, as well as being non-conserved; moreover, eight *BrCOMT* genes were different during the low-temperature condition. This study provides a new insight into how lignin is affected by low temperatures.

## Data Availability

The data presented in this study are available upon request from the corresponding author.

## Supplementary Materials

The following supporting information can be downloaded at https://www.mdpi.com/article/10.3390/genes13112084/s1, Figure S1: Transcriptional relationship between samples. (A) Heatmap of correlation value (R square) of 27 libraries. (B) Principal component analysis based on all of the expressed genes, showing 9 distinct groups of samples. (C) Boxplots of gene expression in each sample, based on the log_10_FPKM of different samples, respectively. Figure S2: MEblue2-related genes and their annotations. (A) Significant GO terms in MEblue2, (B) Significant KEGG in MEblue2, (C) Eigengene expression during each treatment period in MEblue2, heatmap from green to red for expression from high to low, respectively. Figure S3: MElightsteelblue-related genes and their annotations. (A) Significant GO terms in MElightsteelblue, (B) Significant KEGG in MElightsteelblue, (C) Eigengene expression during each treatment period in MElightsteelblue, heatmap from green to red for expression from high to low, respectively. Figure S4: MEsalmon-related genes and their annotations. (A) Significant GO terms in MEsalmon, (B) Significant KEGG in MEsalmon, (C) Eigengene expression during each treatment period in MEsalmon, heatmap from green to red for expression from high to low, respectively. Figure S5: MEbisque4-related genes and their annotations. (A) Significant GO terms in MEbisque4, (B) Significant KEGG in MEbisque4, (C) Eigengene expression during each treatment period in MEbisque4, heatmap from green to red for expression from high to low, respectively. Table S1: qRT-PCR specific primers for *BrCOMT* genes. Table S2: Statistics of Illumina sequencing data. Table S3: List of 8845 genes table for WGCNA. Table S4. List of genes related to lignin synthesis pathway. Table S5. Promoter cis-acting elements analysis of the 29 *BrCOMT* gene.
