# Supplementary figures and images for: Transcriptome Analysis of Chinese Cabbage Provides Insights into the Basis of Understanding the Lignin Affected by Low Temperature
Source: Genes (Basel). 2022 Nov 10;13(11):2084. doi: 10.3390/genes13112084 (PMC9690211; doi:10.3390/genes13112084)

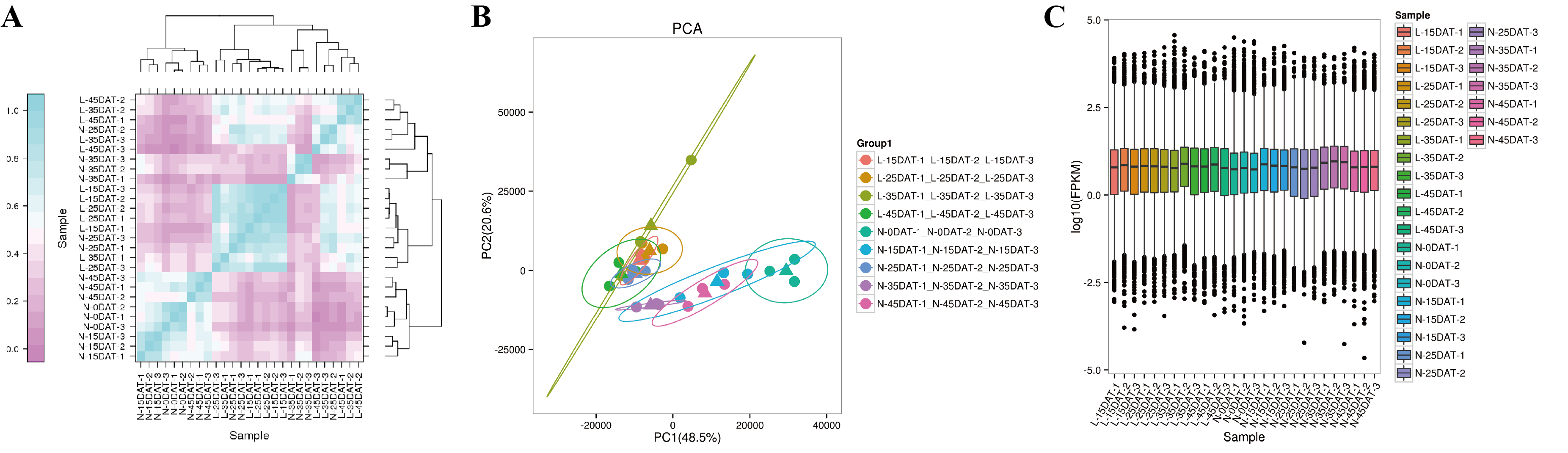

Supplement: Supplementary file 1 [file genes-13-02084-s001.zip › Figure S1.png]

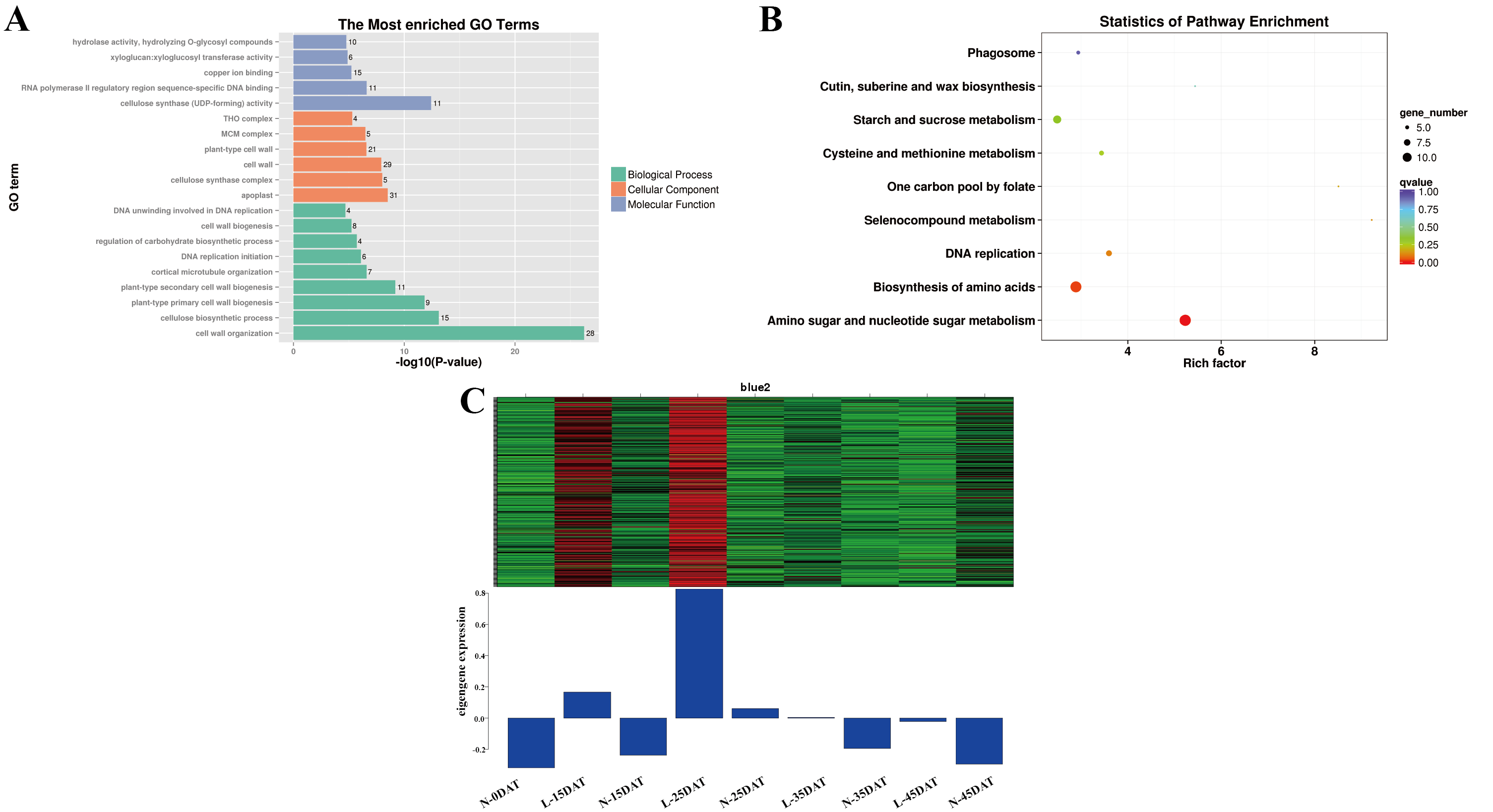

Supplement: Supplementary file 1 [file genes-13-02084-s001.zip › Figure S2.png]

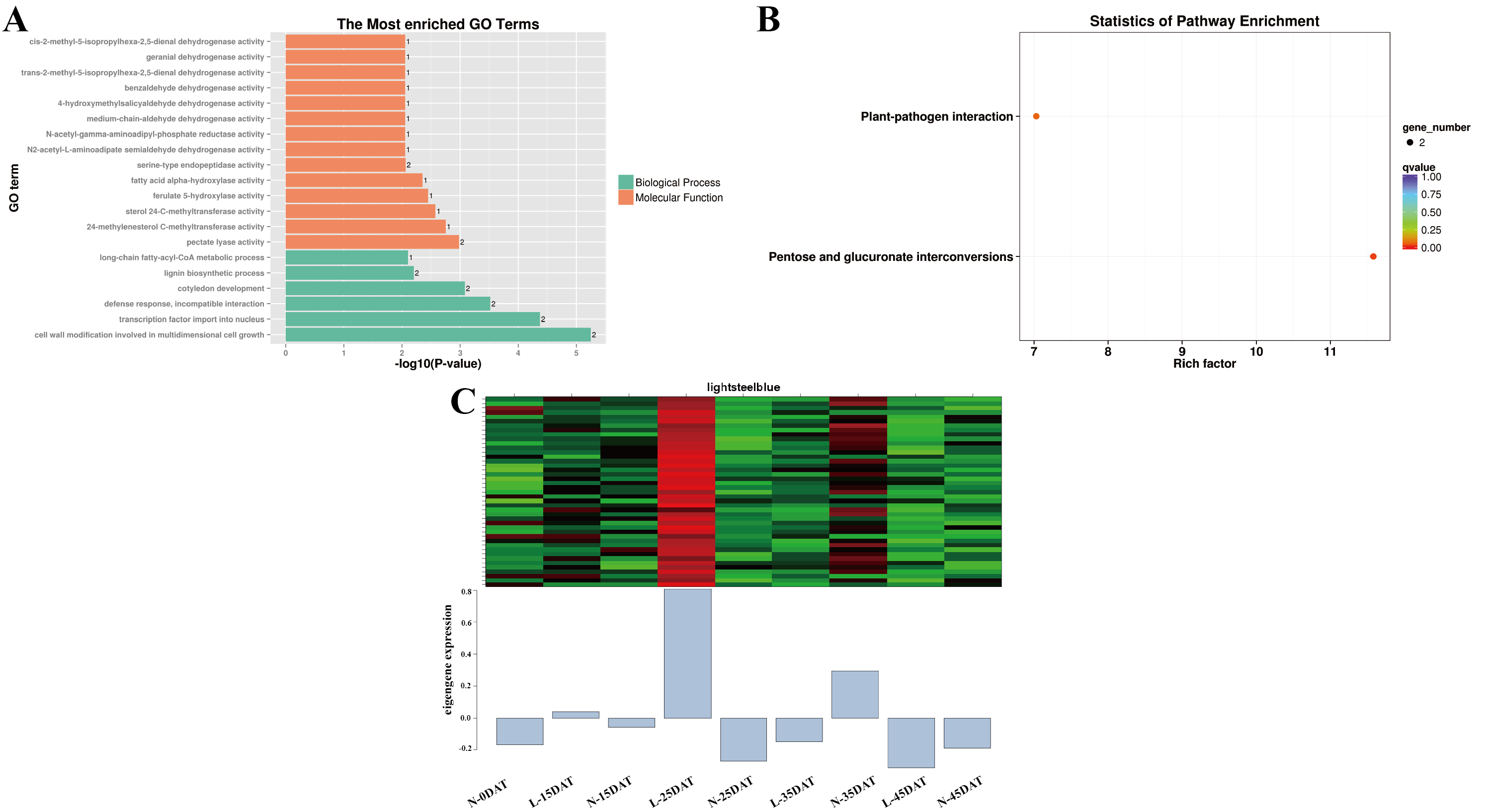

Supplement: Supplementary file 1 [file genes-13-02084-s001.zip › Figure S3.png]

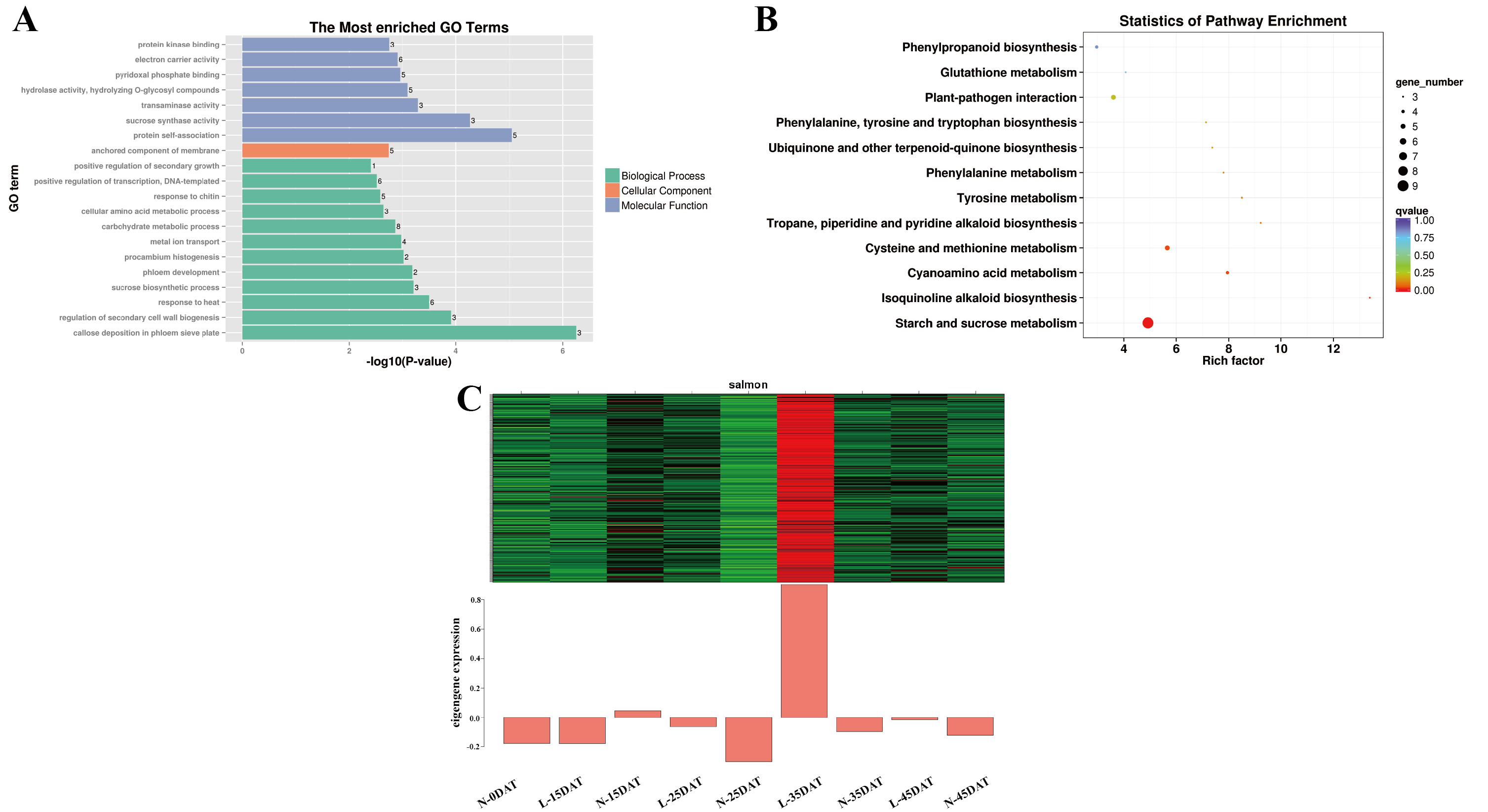

Supplement: Supplementary file 1 [file genes-13-02084-s001.zip › Figure S4.png]

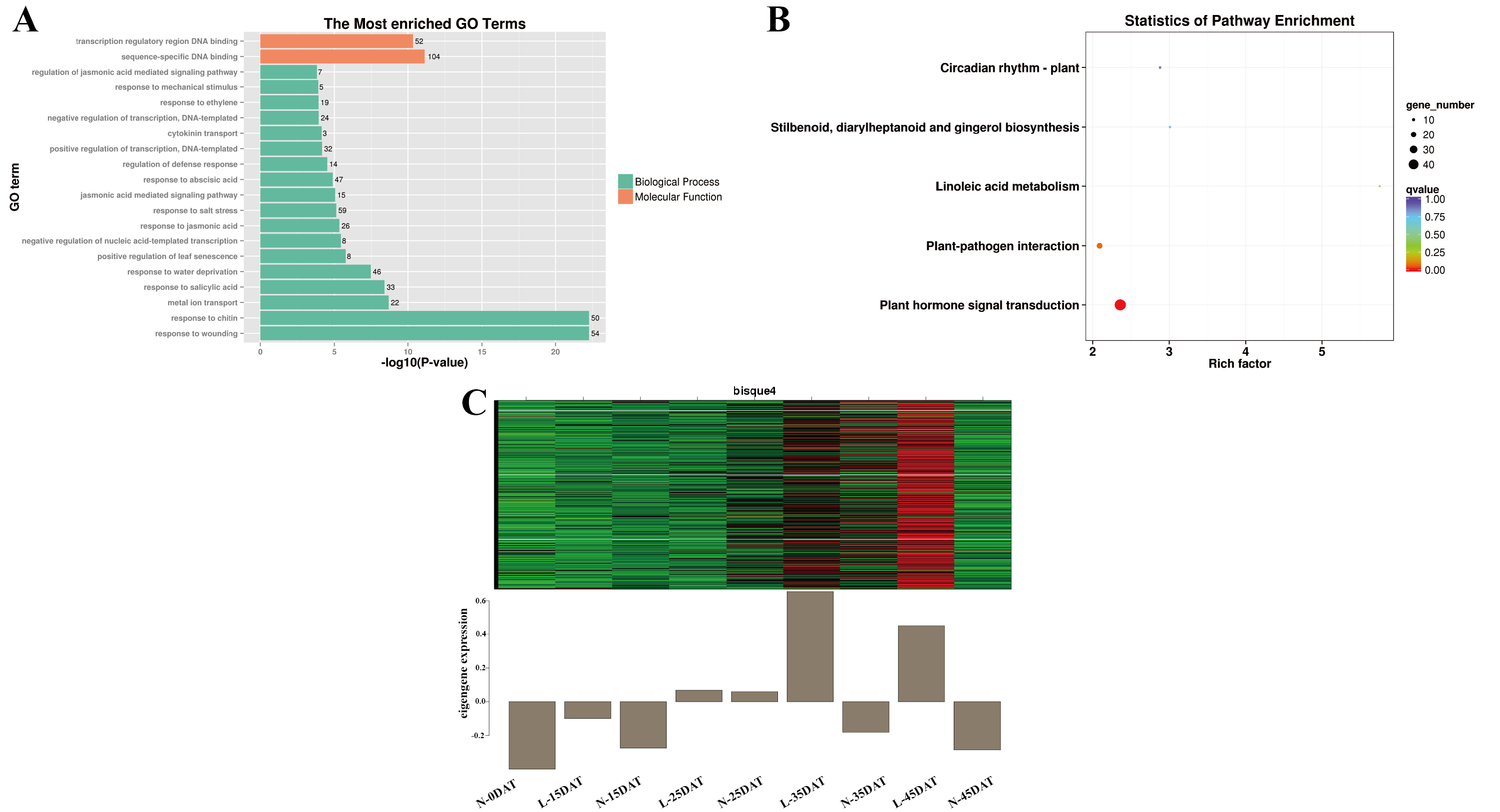

Supplement: Supplementary file 1 [file genes-13-02084-s001.zip › Figure S5.png]
